# Anti-α-amino-3-hydroxy-5-methyl-4-isoxazolepropionic acid receptor encephalitis: A case report

**DOI:** 10.1097/MD.0000000000025694

**Published:** 2021-04-30

**Authors:** Jing Yang, Jichen Du, Jing Zhao, Haichao Liu, Lvming Zhang, Lina Cai, Qi Wang, Bailin Han, Jiangbo Cui

**Affiliations:** a Department of Neurology, Aerospace Center Hospital; b Aerospace Clinic Academy, Peking University Health Science Centre, Beijing, China.

**Keywords:** Anti-α-amino-3-hydroxy-5-methyl-4-isoxazolepropionic acid receptor, case report, neurological disorder

## Abstract

**Introduction:**

: Anti-α-amino-3-hydroxy-5-methyl-4-isoxazolepropionic acid receptor (AMPAR) is a subtype of glutamate receptor that mediates most of the fast excitatory neurotransmission in the brain. Anti-AMPAR encephalitis is an autoimmune-mediated neurological disorder, frequently accompanied by the presence of neoplasms, comprising a spectrum of paraneoplastic syndrome.

**Patient concerns:**

A 56-year-old man was admitted for deterioration in memory and aberrant psychological behaviors, which lasted for at least 20 days

**Diagnosis:**

The patient was diagnosed as anti-AMPAR encephalitis and 4 months later, he was diagnosed with small cell lung cancer.

**Interventions:**

Once diagnosis for anti-AMPAR encephalitis was confirmed, methylprednisolone was prescribed with initial dose 500 mg/d for 14 days until the patient returned to pre-illness state. Then he was discharged with oral treatment with corticosteroids. Following the diagnosis of small cell lung cancer, he received 5 rounds of chemotherapy, including carboplatin and etoposide.

**Outcomes:**

After taken the prescription of Methylprednisolone for anti-AMPAR encephalitis, he returned to pre-illness state and was discharged. In April 21, 2017, after symptoms of respiratory system showed up, he was diagnosed with small cell lung cancer and he eventually died of respiratory failure.

**Conclusion:**

Though progress has been made in recent years in diagnosis and treatment for autoimmune encephalitis, it is challenging to diagnose due to the similarity in clinical findings with other autoimmune or infectious encephalitis. In addition, it is necessary for these patients to regularly have tumor screening, considering AMPAR antibody encephalitis is closely associated with neoplasm, and the incidence of paraneoplastic syndrome is 63% to 70%.

## 1 Introduction

Anti-α-amino-3-hydroxy-5-methyl-4-isoxazolepropionic acid receptor (AMPAR) is a subtype of glutamate receptor that mediates most of the fast excitatory neurotransmission in the brain. Anti-AMPAR encephalitis is an autoimmune-mediated neurological disease, frequently accompanied by the presence of neoplasms, thereby comprising the spectrum of paraneoplastic syndrome.^[1]^ This disease was first reported by Lei et al^[2]^ who analyzed the clinic features of 10 cases with anti-AMPAR encephalitis. Although Wu et al^[3]^ reported the first case of anti-AMPAR encephalitis in China in 2009. Herein, we aimed to report treatment process and follow-up for a patient with anti-AMPAR encephalitis.

## 2 Case presentation

**Figure 1:**
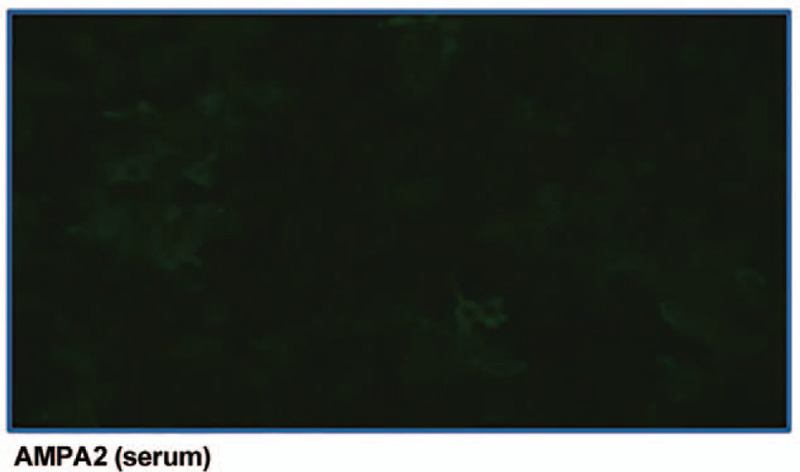
AMPAR antibody in serum.

A 56-year-old man was admitted in November 6, 2016, for deterioration in memory and aberrant psychological behaviors, which lasted for at least 20 days. Besides, 20 days before his admission, he had short-term memory loss, accompanied by anomalies in psychological behaviors, such as emotional disturbances like depression, anxiety fear, irritability, euphoria, apathy, and confusion. There was no fever, headache, dizziness, syncope, seizure, asthenia, or paresthesia during the course of disease. Both computed tomography (CT) scan and cerebrovascular ultrasound revealed no abnormality, and he was initially diagnosed as depression, with no specific treatment prescribed. However, the patient's symptoms showed no sign of relief or improvement. Then, he was transferred to another hospital, and the possibility of autoimmune encephalitis was taken into consideration, EEG was normal, and serum associated-antibody test showed anti-AMPAR encephalitis positive (Fig. 1). Since the disease commenced, he had normal diet and sleep, regular defecation and urination, and his body mass did not significantly change. He had a history of chronic gastritis and gallstones and had undergone cervical disc replacement within 3 years ago. He denied any other diseases, like infection, tumor, and mental disorders.

At admission, he had a blood pressure of 125/80 mm Hg, heart rate of 80 beats/min, slight slowness in response, short-term memory loss, normal long-term memory, and facial paralysis. In addition, MMSE, and MoCA was 21 and 20 respectively.

### 2.1 Auxiliary examination

Blood and urine test: C-reactive protein, coagulation function, tumor marker, and thyroid -stimulating hormone level were all normal; immunoglobulin G of 667 mg/dl (normal range, 751–1560 mg/dl), total hemolytic complement of 54.7 U/ml (normal range, 23.0–52.0 U/ml); thyroglobulin-negative and anti-thyroglobulin antibody-positive, as well as anti-SSB antibody-positive.

Lumbar puncture: cerebrospinal fluid was clear, colorless; intracranial pressure of 150 mmH_2_O, Queckenstedt test (−), Pandy test (+−), red blood cells of 0/HPF, white blood cells (WBCs) of 8/HPF, chloride of 128.1 mmol/L, glucose of 5.63 mmol/L, and antinuclear antibody test showed AMPA2-R (+) (1:10).

**Figure 2:**
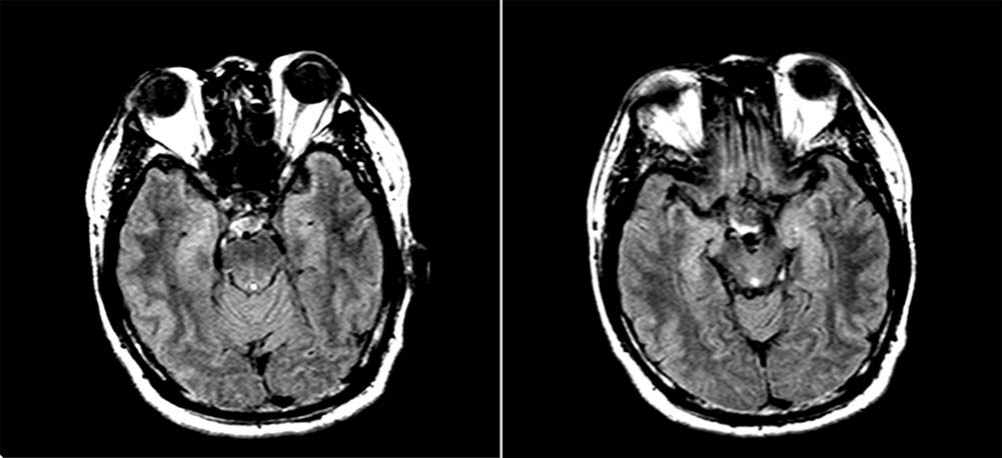
MRI T2/FLAIR shows hippocampal focal T2 hyperintensities with shrinkage in lateral ventricular temporal horn.

Magnetic resonance imaging (MRI) showed hippocampal abnormalities (Fig. 2). CT scan showed pulmonary bullae in upper lobe of both lungs, emphysema, coronary artery calcification. Ultrasonography showed a thick carotid intima-media, plaque formation in carotid artery and gallbladder stones.

### 2.2 Diagnosis and treatment

Combined with symptoms, physical features, and results of auxiliary examination, the diagnosis was anti-AMPAR encephalitis. Methylprednisolone (500 mg/d) was prescribed. After 3-day treatment, his health status was markedly improved, memory enhanced, alertness declined, and he was able to converse normally. After 7-day treatment, he could walk without assistance. After 14-day treatment, his family members found that he had returned to pre-illness state and MMSE was assessed again with a score of 24. Then, he was discharged with oral corticosteroid treatment.

### 2.3 Follow-up

When the patient was discharged, symptoms, such as amnesia or psychological anomalies were not observed any more.

**Figure 3:**
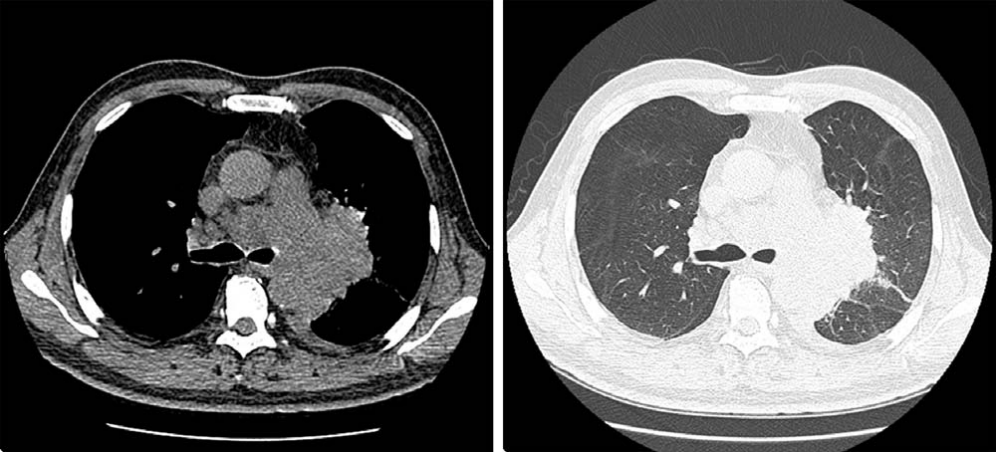
CT scan shows bronchial soft tissue shadow on the posterior segment of the left upper lung apex, unclear borders, obstruction of the posterior segment of the upper lung apex, and enlarged lymph nodes in the mediastinum.

**Figure 4:**
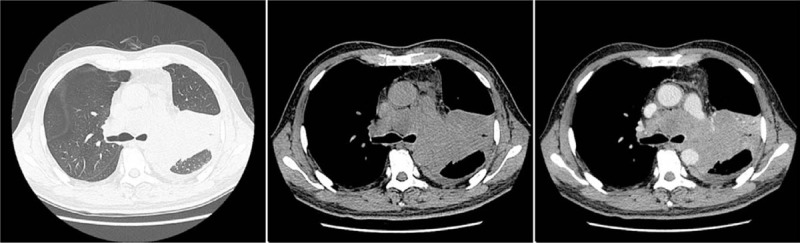
Progressive enlargement of the left upper lung lesion, enlarged lymph nodes in the mediastinum with the enhancing lesion.

In March 2017, 4 months after initiation of the disease, he had cough with a small amount of white sputum, and bloodstains could be observed in the sputum, accompanied by chest tightness. At the same time, he underwent CT scan. After comparing the results in November 10, 2016, we found inflammatory changes in the left upper lobe. After undergoing nonsteroidal anti-inflammatory drugs, another CT was carried out, that revealed a left-sided pleural effusion, and other lesions were the same as the latest one (Fig. 3). In April 21, 2017, he was diagnosed with small cell lung cancer, and immunohistochemistry results were as follows: TTF-1(+), CgA(−), Syn(+), CK(+), CD56(+), Ki-67(+75%), P63(−). In April 28, 2017, physical examination revealed that there was an enlarged lymph node nearby right clavicle with a diameter of about 3 cm. Testing of the levels of related tumor markers in blood suggested gastrin-releasing peptide level of 5244.82 pg/mL (range, 0—50.00 pg/mL) and neuron specific enolase level of 133.75 ng/mL (range, 0—20.00 pg/mL). Swollen lymph nodes were found on the right supraclavicular fossa and both sides of the neck. In May 03, 2017, he underwent another CT scan (Fig. 4), which indicated that the lesion in left superior lung lobe was further intensified, which was accompanied by more pleural effusion on left side and larger mediastinal lymph nodes than the last CT scan; meanwhile, pulmonary arteries and unnamed veins and superior vena cava in both lungs were involved. Whole-body bone scintigraphy was performed, and revealed that there was a concentrated radioactive focus on the left upper femur. Under that circumstance, he received carboplatin and etoposide (CE) chemotherapy. From April to August 2017, a total of 5 rounds of chemotherapy were performed. He eventually died of respiratory failure in January 2018.

## 3 Discussion

Autoimmune encephalitis is a challenging clinical diagnosis due to the similarities in the clinical, imaging, and laboratory findings with autoimmune and infectious encephalitis. AMPAR is a transmembrane ionic glutamate receptor, which belongs to a chemically gated ion channel receptor and a tetramer composed of GluR1-GluR4 subunits. It is mainly distributed in the hippocampus, and other brain regions in the limbic system.^[4,5]^

Under normal physiological conditions, AMPAR is impermeable to calcium ions (Ca^2+^). It also can cause sodium ion influx to further intensify depolarization of the post-synaptic membrane, forming rapid excitatory post-synaptic potentials, thereby participating in the fast excitatory synaptic transmission in the autonomic nervous system.^[6]^ However, the emergence of AMPAR antibodies may cause excessive activation of AMPAR, and then more GluR2 subunits are phosphorylated, resulting in internalization and reduction of GluR2 in the post-synaptic membrane, and continuous Ca^2+^ influx triggers a series of abnormalities in both neurological functions and information transmission.^[7]^

Additionally, the continuous influx of Ca^2+^ can activate intracytoplasmic proteases, phospholipases, ATPases, etc, causing nerve cells to swell and apoptosis.^[8]^ Surface expression of AMPA receptors in hippocampal neurons is regulated by N-ethylmaleimide-sensitive fusion dependent mechanism. The direct damage of antibodies to neurons can be reflected in MRI, and abnormal signals are typically manifested in the hippocampus, cerebellum, cerebral cortex, and basal ganglia.^[9,10]^

Secondly, increased permeability to Ca^2+^ also could lead to dysfunction of long-term potentiation (LTP) in synapses.^[11]^ Synaptic plasticity, comprised of LTP and long-term depression, is the foundation of learning and memory, playing a pivotal role in enhancement of central nervous system.^[12]^ With the emergence of AMPAR antibody, the expression of GluR2 subunit is reduced, Ca^2+^ permeability is increased, the length, width, and density of LTP are reduced, and the number of functional synapses is reduced, resulting in a decline in memory and cognitive function.^[13]^ AMPAR antibody directly causes loss and damage to hippocampal neurons. Moreover, it leads to the dysfunction of synaptic plasticity, causing clinical symptoms to be characterized by memory and cognitive dysfunction.

Additionally, the continuous influx of Ca^2+^ can also change the synaptic current, triggering abnormal synchronous discharge of neurons, thus, epilepsy could be a common clinical symptom in such patients. However, the patient in the current report had no epilepsy, and we found no evidence of abnormal neuronal discharge.

It is currently well-known that dysregulation of glutamate transmitters is one of the indispensable causes of depression.^[14]^ AMPA receptor stimulation is responsible for the antidepressant effects of ketamine and mGlu2/3 receptor antagonists in 2 different ways: one is via glutamate release immediately after the injection and the other is via the upregulation of AMPA receptor expression and neurotransmission.^[15]^ The emergence of AMPAR antibody not only could cause depression, but also might prevent antidepressant effects of other drugs. A recent study explained this mechanism, in which the initiation of AMPAR can activate mitogen-activated protein kinase (MAPK) cascade, increase the volume and phosphorylation of extracellular signal-regulated kinases, and ultimately lead to increased expression of receptor tyrosine kinases, thereby enhancing synaptic plasticity and the connection between synapses to increase nerve excitability, and improve depressive-like symptoms. If AMPAR antibodies reduce the amount of AMPAR and decrease phosphorylation, this could lead to depression. In the present report, we noticed that the patient was misdiagnosed as depression.

We concluded that the impairment of the functions and mechanisms of AMPAR subunits determines the clinical symptoms of patients, including amnesia, depression, limbic system disorder, and seizure. When encountering patients with the above-mentioned clinical manifestations, AMPAR antibody encephalitis should be highly suspected. The diagnosis requires detection of cerebrospinal fluid and serum AMPAR antibody. Once the antibody is detected, the diagnosis can be made.^[2,16]^ Neuronal culture experiments confirmed that after a few days of AMPAR antibody clearance, the number of receptors can return to normal.^[2]^ In the current case, the patient was tested for serum antibodies twice, the result of the first test was positive with a titer of 1:100, and after immunotherapy, his condition was notably improved, and the antibody titer was reduced to 1:10. This indicated that antibody titer may be used as an important indicator for disease severity and follow-up.

Because AMPAR antibody encephalitis is often associated with tumor or with other autoimmune antibodies, its prognosis is worse than other types of autoimmune encephalitis and has a tendency to relapse. This patient was diagnosed with small cell lung cancer at 4 months after the onset of the disease. Although no encephalitis recurred during the process, he eventually died from a malignant tumor. Under this circumstance, except for application of antibody titer in follow-up, patients also need to undergo regular tumor screening. According to prior studies, AMPAR antibody encephalitis is closely associated with neoplasm (lung, thymoma, breast, and ovary), and the incidence of paraneoplastic syndrome is 63% to 70%.^[1,17]^ Therefore, it is recommended that patients aged older than 18 years old should undergo regular tumor and onconeuronal antibodies screening.^[18]^ Early detection of a potential tumor or recurrence of a tumor can reduce its relapse rate and improve prognosis.^[19]^ If the result of the first tumor screening is negative, it should be rechecked after 3 to 6 months, with follow-up screening every 6 months for 4 years.^[20,21]^ The patient in the current report had a negative result of tumor screening when he was admitted to the hospital, and there was no obvious lesion on CT scan. However, respiratory symptoms appeared 4 months after the onset of the disease, and the lung cancer was diagnosed by pathological examination, while he eventually died of respiratory failure. The follow-up findings of this patient suggested that for those patients with AMPAR antibody encephalitis who have recovered well after treatment, regular follow-up examinations should be performed to detect potential tumor as early as possible.

With the development of antibody detection technology, AMPAR antibody encephalitis has been increasingly recognized. Its clinical manifestations can be explained by the pathological changes due to AMPAR antibody formation, and the antibody titer is related to therapeutic effect. It is noteworthy that AMPAR antibody encephalitis is closely associated with tumors, thus, tumor-related screening is essential for patients with AMPAR antibody encephalitis.

## Author contributions

**Conceptualization:** Jichen Du.

**Data curation:** Jing Zhao, Haichao Liu, Lvming Zhang, Lina Cai.

**Formal analysis:** Jing Yang, Jing Zhao, Lvming Zhang.

**Investigation:** Jing Zhao, Bailin Han.

**Methodology:** Jing Yang, Jichen Du.

**Project administration:** Jichen Du.

**Resources:** Haichao Liu.

**Software:** Lina Cai, Qi Wang.

**Supervision:** Qi Wang.

**Validation:** Jiangbo Cui.

**Writing – original draft:** Jing Yang, Jichen Du.

**Writing – review & editing:** Jing Yang, Jing Zhao, Haichao Liu, Lvming Zhang, Lina Cai, Qi Wang, Bailin Han, Jiangbo Cui, Jichen Du.
